# Quetiapine Versus Haloperidol in the Management of Hyperactive Delirium: Randomized Controlled Trial

**DOI:** 10.1007/s12028-024-01948-w

**Published:** 2024-04-01

**Authors:** Tamer Zakhary, Islam Ahmed, Ibrahim Luttfi, Mina Montasser

**Affiliations:** 1 Critical Care Medicine Department, Faculty of Medicine, Alexandria University, Alexandria, 21111 Egypt; 2 Public Health and Community Medicine Department, Faculty of Medicine, Suez-Canal University, Ismailia, Egypt; 3 Primary Health Care and Health Education Department, Faculty of Medicine, Gezira University, Wad Medani, Sudan; 4 Emergency Medicine Department, Faculty of Medicine, Alexandria University, Alexandria, Egypt; 5 Pharmacy Practice and Clinical Pharmacy Department, King Salman International University, South Sinai, Egypt

**Keywords:** Delirium, Hyperactive, Antipsychotics, Haloperidol, Quetiapine

## Abstract

**Background:**

In the population of patients in the intensive care unit (ICU), most studies compared the use of atypical antipsychotics, such as quetiapine, with the use of traditional haloperidol in patients with delirium of various forms and etiologies. The role of such agents in patients with hyperactive delirium is not fully understood. This study compares the effectiveness of quetiapine with haloperidol in treating the hyperactive form of delirium in terms of their effects on the Delirium Rating Scale-Revised-98 (DRS-R-98), length of stay in the ICU, and mortality in critically ill patients.

**Methods:**

One hundred adult patients diagnosed with hyperactive delirium were randomly assigned to receive either oral quetiapine (25–50 mg/day) or haloperidol (1–2 mg/day). The response, defined as “a DRS-R-98 severity score reduction from baseline of 50% or more” and a DRS-R-98 severity score of 12 or less without relapse, was the primary outcome.

**Results:**

The mean age of all patients was 68 ± 6 years. The study population’s overall response rate was 92%. Response rates for the two groups were remarkably equal (*p* = 0.609). Secondary outcomes were comparable in both groups, such as ICU mortality (*p* = 0.496), in-hospital mortality (*p* = 0.321), in-hospital stay (*p* = 0.310), and the need for mechanical ventilation (*p* > 0.99). But the quetiapine group showed a statistically reduced mean ICU stay (10.1 ± 2.0 vs. 11.7 ± 2.6 days, *p* = 0.018) and increased sleeping hours per night (*p* = 0.001).

**Conclusions:**

Quetiapine may be equally as effective as haloperidol in treating the symptoms of hyperactive delirium in critically ill patients, with no mortality benefit.

## Introduction

Delirium, a newly recognized but increasingly prevalent complication in the intensive care unit (ICU), casts a shadow over a critically ill patient. Defined in the Diagnostic and Statistical Manual of Mental Disorders, Fifth Edition, as a “sudden deterioration in attention, awareness, and cognition” not caused by preexisting brain disorders but rather by underlying medical conditions, delirium significantly impacts patient outcomes [1, 2].

A staggering meta-analysis of 42 studies, encompassing 16,595 critically ill patients, revealed a shockingly high prevalence of delirium: 31.8% [3]. This rate is significantly higher than the general population, highlighting the alarmingly increased risk of delirium in the ICU setting. The prevalence varied greatly, ranging from a low of 9.2% for severely ill surgical patients who were not mechanically ventilated to a staggering 91% for oncology patients receiving mechanical ventilation [4]. This vast range underscores the complex interplay of factors contributing to delirium, including preexisting medical conditions, the severity of illness, and specific treatment interventions.

In liaison consultation, managing delirium is critical for patient well-being. Traditionally, butyrophenone antipsychotics, such as haloperidol, are the first-line treatment. However, these medications are associated with significant side effects, including extrapyramidal symptoms (EPS) and severe sedation [5]. Haloperidol’s EPS have been a major concern for delirium management. Studies suggest that up to 30% of patients with delirium taking a daily doses of 5–15 mg of haloperidol experience EPS, compared with a lower rate observed in patients receiving atypical antipsychotics [6]. These adverse effects can be distressing for patients, negatively impact their recovery, and necessitate dose adjustments or discontinuation of the medication.

With the emergence of new evidence and concerns about haloperidol’s side effects, the focus has shifted toward safer and more effective treatment options. Atypical antipsychotics, such as quetiapine and risperidone, are increasingly being recommended because of their lower risk of EPS and improved tolerability.

A dibenzothiazepine derivative with a novel and distinctive pharmacologic profile is quetiapine. It is increasingly being recognized as a promising alternative to haloperidol for managing delirium, particularly because of its favorable side effect profile. One of the key pathophysiological mechanisms of delirium is hyperactivity in the limbic system. This brain region plays a critical role in emotion, memory, and processing sensory information. In delirium, this area becomes overactive, leading to the characteristic symptoms of confusion, agitation, and cognitive impairment. Quetiapine’s unique mechanism of action offers potential benefits in managing this limbic hyperactivity. Unlike haloperidol, which blocks dopamine D2 receptors broadly throughout the brain, quetiapine specifically targets these receptors in the mesolimbic pathway, a key circuit within the limbic system. This targeted approach helps to regulate limbic activity without causing the widespread side effects seen with haloperidol [5].

The objective of this study was to compare the effectiveness of quetiapine with haloperidol in treating the hyperactive form of delirium in terms of their effects on the Delirium Rating Scale-Revised-98 (DRS-R-98), length of stay in the ICU, and mortality in critically ill patients.

## Methods

**Fig. 1:**
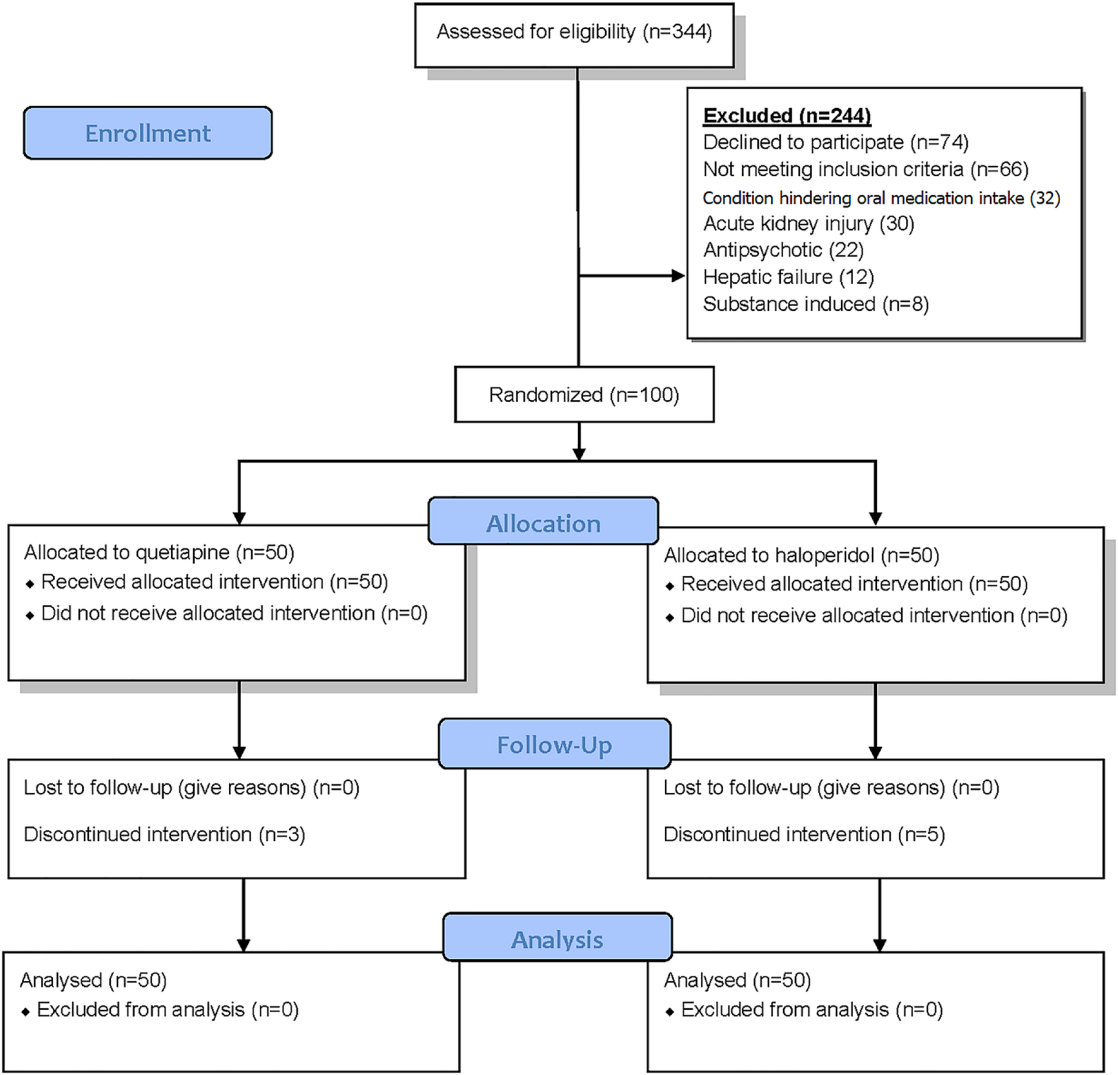
Flow diagram of the study

In this study, 344 patients were assessed for enrollment. One hundred adult patients (*n* = 100) were enrolled and involved in the final analysis. All patients were diagnosed with a hyperactive form of delirium during their ICU stay using the confusion assessment method for the ICU tool [7] and Richmond Agitation Sedation Scale at Alexandria University Hospitals in Egypt from April to July 2023. Patients with suspected substance-induced delirium; previous use of antipsychotics; known allergies or intolerances to the study drugs; pregnancy or breast feeding; acute renal injury, hepatic failure, or any condition hindering oral medication intake; recent central nervous system pathology hemorrhage or stroke; and head trauma were excluded. The flow diagram is illustrated using Consolidated Standards of Reporting Trials 2010 in Fig. 1.

Written informed consent was obtained from the patient’s legal guardian in a private room beside the ICU following approval by the research ethics committee, the Department of Critical Care Medicine of the Faculty of Medicine at Alexandria University, and after thorough explanation of the benefits and risks of the study interventions. This study’s protocol was registered on ClinicalTrials.gov (identifier: NCT05690698).

At the time of enrollment, patients were subjected directly to a complete history and demographic data collection, a physical examination, routine laboratory investigations, a hormonal profile, and brain computed tomography. Possible risk factors and assessment of delirium were collected from their recorded data using mnemonics (IWATCHDEATH: I, infections; W, withdrawal; A, acute metabolic; T, toxins/drugs; C, central nervous system pathology; H, hypoxia; D, deficiencies; E, endocrine; A, acute vascular; T, trauma; and H, heavy metals) [8]. The DRS-R-98 severity score [9] was calculated at time of diagnosis (day 1) of delirium.

In this double-blind randomized controlled trial (RCT), along with the allocation concealment, patients were randomly assigned using a computer sheet (randomizer.org) into two groups with a 1:1 allocation ratio. Double blindness was achieved using overencapsulation. The quetiapine group (*n* = 50) received oral or nasogastric quetiapine (25–50 mg/day) according to their symptoms of agitation. The haloperidol group (*n* = 50) received oral or nasogastric haloperidol (1–2 mg/day) according to their symptoms of agitations. Patients were followed up and received their standard care during their hospital stay. DRS-R-98 severity score was followed up two times (day 3 and day 7). The DRS-R-98 was measured by two independent attending physicians, one of them was not aware of the goal of the study. The participation of any patient was terminated if any adverse effect developed, if no enteral medications were ordered, or if discharge occurred before 7 days. All patients enrolled were included in the final analysis (intention-to-treat analysis).

The primary outcome of this study was the response rate (defined as a reduction of the DRS-R-98 severity score from its baseline of 50% or more and a DRS-R-98 severity score of 12 or less without relapse). The secondary outcomes were ICU stay, hospital length of stay, the need for mechanical ventilation, daily sleeping hours, ICU mortality, and in-hospital all-cause mortality rates. Sleeping hours were measured using a subjective method via nursing observation (observing sleep–wake patterns, recording periods of quiet, and noting disruptions). Any fraction of hour was calculated as 1 h.

### Statistical Analyses

The minimum required sample size was calculated using G*Power software (3.1.9.4) based on previous pilot-trial effect size of 0.653, an α error of 5%, and an expected power of 95%. Data were fed to the computer and analyzed using IBM SPSS software package version 24.0 (IBM Corp, Armonk, NY). Qualitative data were described using numbers and percentages. Quantitative data were described using mean and standard deviation or median and interquartile range. The tests used were Student’s *t* test, Mann–Whitney *U* test, and *χ*^2^ test with Mcnemar and Bonferroni corrections. The significance of the obtained results was judged at the 5% level. No patients were excluded from the final analysis, even if treatment was changed because of inefficacy or if adverse effects were reported (intention-to-treat analysis).

## Results

In this RCT, 100 patients with hyperactive delirium were enrolled and randomly assigned into two groups (the quetiapine group and the haloperidol group). Regarding baseline characteristics, the mean age of all patients was 68 ± 6 years. Sixty percent of all enrolled patients were female. The most prevalent risk factors for delirium were malnutrition (58%), infections (50%), and electrolyte disturbances (44%). There were no statistically significant differences between the two groups in their baseline characteristics (Table 1).

**Table 1:** Baseline characteristics of all enrolled patients

Variables	Overall (*N* = 100)		Quetiapine (*n* = 50)		Haloperidol (*n* = 50)		*p* value
	*n*	Percent	*n*	Percent	*n*	Percent	
Female sex	60	60.0	36	72.0	24	48.0	0.148
Cause of admission							
Cardiac disease	60	60.0	26	52.0	35	68.0	0.387
Infections	30	30.0	16	32.0	14	28.0	> 0.99
Trauma	24	24.0	14	28.0	10	20.0	0.742
Chest disease	18	18.0	12	24.0	6	12.0	0.463
Risk factors							
Malnutrition	58	58.0	30	60.0	28	56.0	> 0.99
Infections	50	50.0	28	56.0	22	44.0	0.572
Electrolyte disturbance	44	44.0	22	44.0	22	44.0	> 0.99
Hypoxia	36	36.0	18	36.0	18	36.0	> 0.99
Hemodynamics	30	30.0	18	36.0	12	24.0	0.538
Trauma	24	24.0	14	28.0	10	20.0	0.742
CNS pathology	18	18.0	12	24.0	6	12.0	0.463
Endocrine	6	6.0	2	4.0	4	8.0	0.552
Age (y)	68 ± 6		68 ± 6		69 ± 6		0.869
APACHE II score	24.7 ± 2.9		24.2 ± 3.16		24.2 ± 2.71		0.236
Hb (g/dL)	10.3 ± 0.8		10.4 ± 0.77		10.1 ± 0.75		0.217
WBCs (× 10^3^/µL)	7.9 ± 4.7		7.98 ± 5.1		7.88 ± 4.3		0.936
PLTs (× 10^3^/µL)	239 ± 110.5		247 ± 123.9		230 ± 97.1		0.586
Na (mEq/L)	130 ± 5.0		131 ± 4.5		129 ± 5.5		0.358
K (mmol/L)	3.3 ± 0.3		3.3 ± 0.3		3.2 ± 0.4		0.401
DRS-R-98 (day 1)	29.0 (6.0)		28.0 (6.0)		30.0 (7.0)		0.502
Sleeping hours (day 1)	1.9 (0.6)		1.8 (0.5)		2.2 (0.6)		0.001*

Data are expressed as mean ± standard deviation or median (interquartile range)

Malnutrition: documented or possible hypovitaminosis (B_12_, niacin, thiamine)

Infections: sepsis without encephalitis or meningitis

Electrolyte disturbance: hypokalemia

Hypoxia: acute hypoxia, chronic lung disease

Hemodynamics: persistent hypotension, hypertensive emergency

Trauma: any trauma except head trauma

CNS pathology: vasculitis, seizures, no hemorrhage, or ischemic stroke

Endocrine: diabetes or thyroid disease

APACHE II, Acute Physiology, Age, and Chronic Health Evaluation version II score, CNS, central nervous system, DRS-R-98, Delirium Rating Scale-Revised-98 severity score, Hb, hemoglobin, K, potassium level, Na, sodium level, PLT, platelet count, WBC, white blood cell count

^*^All *p* values are significant when *p* ≤ 0.05

**Fig. 2:**
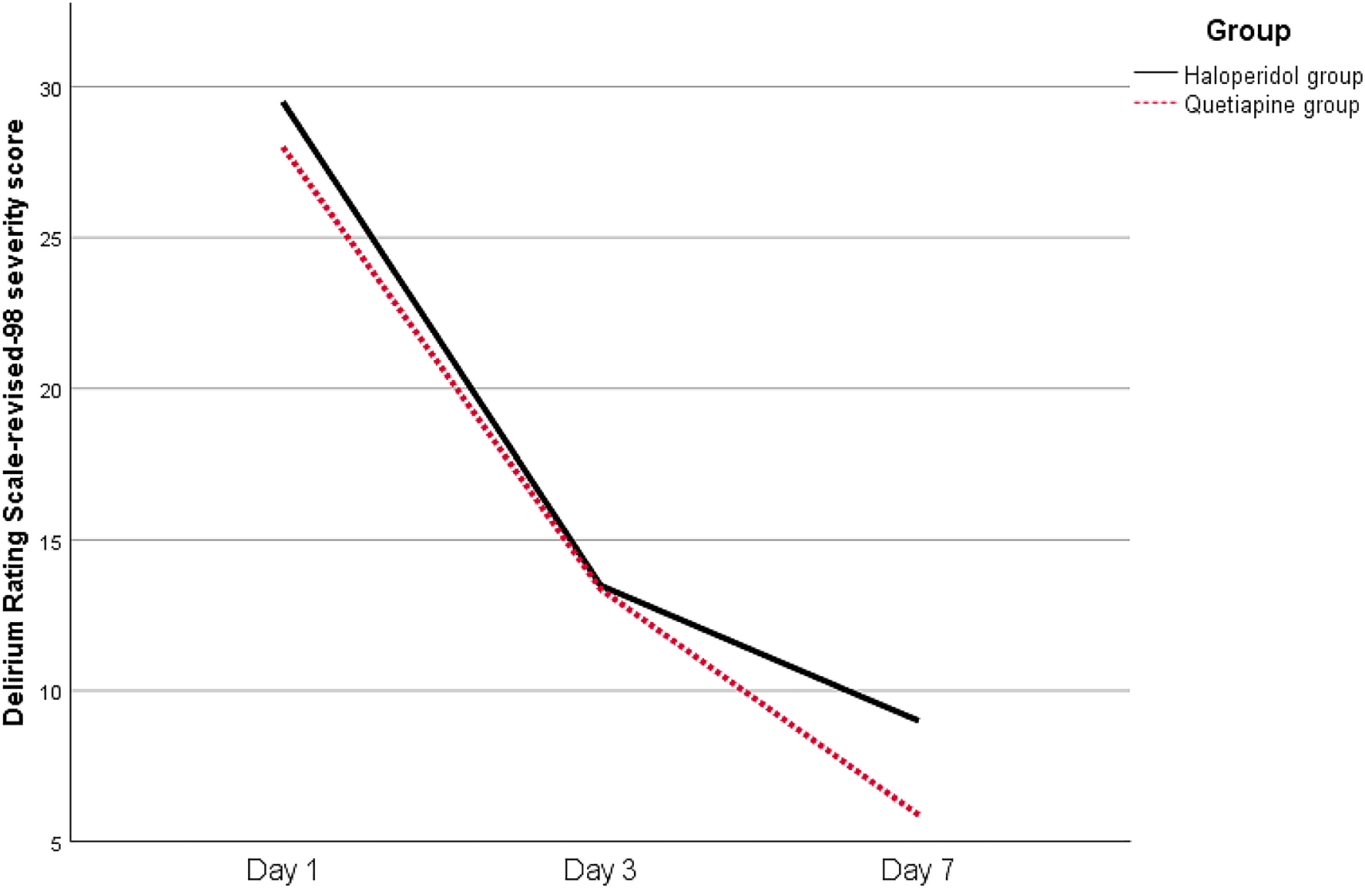
Trend of the median DRS-R-98 severity score over time for the two studied groups. DRS-R-98, Delirium Rating Scale-Revised-98

In the current trial, the median DRS-R-98 severity scores for the two groups were comparable at days 1 and 3, with no statistically significant differences between them (*p* = 0.502 and *p* = 0.946, respectively). When compared with haloperidol, the quetiapine group had significantly reduced median DRS-R-98 severity scores at day 7 (5 vs. 9, *p* < 0.001) (Table 2; Fig. 2). The study population’s overall response rate was 92%. The response rates for the two groups were remarkably similar (88% for the haloperidol group and 96% for the quetiapine group, *p* = 0.609). Regarding safety and adverse events, five patients in the haloperidol group showed QT prolongation (*n* = 1) and extrapyramidal side effects (*n* = 4). Three patients in the quetiapine group developed QT prolongation.

**Table 2:** The measured study outcomes of all enrolled patients

Variables	Overall (*N* = 100)		Quetiapine (*n* = 50)		Haloperidol (*n* = 50)		*p value*
	*n*	Percent	*n*	Percent	*n*	Percent	
DRS-R-98 (day 3)	13.0 (5.0)		13.0 (6.0)		13.0 (6.0)		0.946
DRS-R-98 (day 7)	7.0 (4.0)		5.0 (4.0)		9.0 (5.0)		< 0.001*
Sleeping hours (day 3)	3.25 (1.3)		3.4 (1.0)		2.7 (1.3)		0.038*
Sleeping hours (day 7)	4.45 (3.1)		6.0 (3.6)		3.5 (2.3)		< 0.001*
Response rate ^a^	92	92.0	48	96.0	44	88.0	0.609
Need for MV	38	38.0	18	36.0	20	40.0	> 0.99
ICU mortality	22	22.0	8	16.0	14	28.0	0.496
In-hospital mortality	24	24.0	8	16.0	16	32.0	0.321
ICU stay (days)	11.0 ± 2.4		10.1 ± 2.0		11.7 ± 2.6		0.018*
In-hospital stay (days)	15.0 ± 3.7		14.3 ± 3.4		15.4 ± 4.0		0.310

Data expressed as mean ± standard deviation or median (interquartile range)

DRS-R-98, Delirium Rating Scale-Revised-98 severity score, ICU, intensive care unit, MV, mechanical ventilation

^*^All *p* values are significant when *p* ≤ 0.05

^a^Response rate: reduction of the DRS-R-98 severity score from its baseline of 50% or more and a DRS-R-98 severity score of 12 or less without relapse

**Fig. 3:**
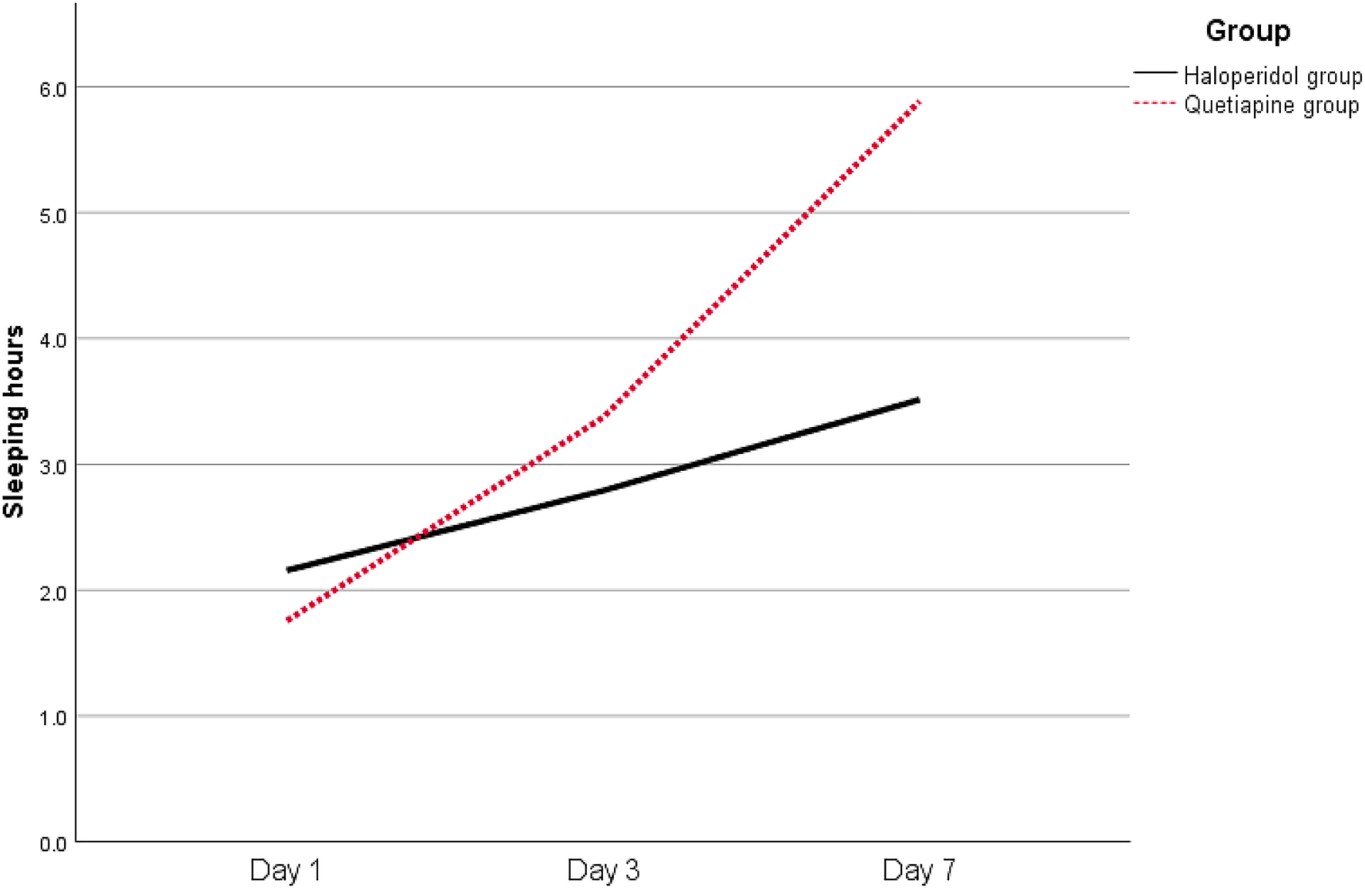
Trend of the median sleeping hours over time for the two studied groups

On the first day, the haloperidol group had longer median sleeping hours than the quetiapine group (2.2 vs. 1.8 h), and these differences were statistically significant (*p* = 0.001). Then, on days 3 and 7, respectively, the quetiapine group had substantially longer median sleeping hours than the haloperidol group (3.4 vs. 2.7 h, *p* = 0.038; and 6 vs. 3.5 h, *p* < 0.001) (Fig. 3).

The mean duration of ICU stay for all patients was 11.0 + 2.4 days. The mean duration of ICU stay for the quetiapine group (10.1 ± 2.0 days) was significantly lower than that of the haloperidol group (11.7 ± 2.6 days; *p* = 0.018). Without statistically significant variations, both groups showed comparable means for the duration of hospital stay (*p* = 0.310; Table 2).

## Discussion

The majority of research in critically ill patients compared haloperidol with atypical medications, such as quetiapine, in mixed populations of patients with delirium who were hypoactive, hyperactive, and mixed in nature, coming from mixed etiologies and comorbidities. Which medications may be more effective in critically ill patients with hyperactive delirium remain unclear. This comparative study compares the effectiveness of quetiapine with haloperidol as a control in treating the hyperactive form of delirium in terms of their effects on DRS-R-98 score, length of stay, and mortality. According to our findings, the study sample’s clinical response rate was 92%. The response rates for the two groups were comparable (88% for haloperidol and 96% for quetiapine, *p* = 0.609).

According to the study’s secondary outcomes, there were no statistically significant differences between the two groups’ mechanical ventilation needs (*p* > 0.99), hospital stay (*p* = 0.310), ICU mortality (*p* = 0.496), or in-hospital mortality (*p* = 0.321). In terms of ICU stay, there was a statistically significant difference between the haloperidol group (11.7 ± 2.6 days) and the quetiapine group (10.1 ± 2.0 days) (*p* = 0.018).

A single-blind RCT was undertaken by Grover et al. [10]. A total of 63 patients with delirium were enrolled, and 87% of them had the hyperactive form. Haloperidol (0.25–1.25 mg/day) was administered to 32 individuals, whereas quetiapine (12.5–75 mg/day) was given to another 31 patients. Both groups were evaluated at the beginning and 6 days later. Initially, there were no statistically significant differences between the means of the DRS-R-98 severity scores for the two groups (24.81 ± 2.19 for haloperidol, 25.48 ± 3.60 for quetiapine). Both groups showed comparable means at days 3 (*p* = 0.26) and 7 (*p* = 0.679) following the follow-up. At day 6, the response rates for the two groups were nearly identical (68.75% for haloperidol, 67.74% for quetiapine), with no statistically significant differences between them (*p* = 0.93) [10].

In a double-blind RCT, Maneeton et al. [11] examined 52 medically ill patients with hyperactive delirium. The most prevalent risk factors for delirium were trauma, fluid-electrolyte imbalance, and infections. Patients were given either quetiapine or haloperidol at random allocation. Baseline DRS-R-98 scores for the two groups (haloperidol, 29.7 ± 4.6 and quetiapine, 29.0 ± 4.4) were comparable (*p* = 0.23). After 7 days, the mean DRS-R-98 score differences between haloperidol (− 21.7 ± 6.7) and quetiapine (− 22.9 ± 6.9) were comparable but not significantly different (*p* = 0.59). On day 7, there were no noticeable differences in the response rates for haloperidol (78.5%) and quetiapine (79.5%) (*p* = 0.97) [11].

In an open label trial, 12 patients with delirium were evaluated by Sasaki et al. [5]. Patients received quetiapine (25–50 mg/day). The Japanese version of the DRS was used to evaluate patients. The mean duration of treatment until remission was 4.8 ± 3.5. The baseline mean Japanese version of the DRS score was 18.1 ± 4.2, and it was changed to 9.3 ± 1.6 after remission [5].

Omura et al. [12] evaluated 24 older patients who had been given a Diagnostic and Statistical Manual of Mental Disorders, Fourth Edition, delirium diagnosis. Initial dosages of 25–50 mg/day quetiapine were given to patients, and then subsequent dosages were adjusted based on their clinical responses. Initially, the mean DRS score was 18.1 ± 3.7. The mean score was 8.9 ± 3.9 on day 7 of the quetiapine treatment, which is a statistically significant difference (*p* < 0.001). The clinical response at day 7 was recorded in 75% of the study population [12].

In the present trial, the median number of sleeping hours on day 1 was considerably lower in the quetiapine group than in the haloperidol group (*p* = 0.001). At days 3 and 7, quetiapine had significantly higher median sleeping hours than haloperidol (*p* = 0.038 and *p* = 0.001, respectively). In contrast to these findings, the Maneeton et al. [11] study found no significant differences in the mean sleeping hours between the haloperidol and quetiapine groups at day one (*p* = 0.26). On day 7, results showed an increase in the mean sleeping hours in both groups, with no discernible difference between them (*p* = 0.74) [11].

In the Sasaki et al. [5] study, the quetiapine-treated group did not exhibit severe daytime somnolence or sedation. In the Kim et al. [13] trial, quetiapine was well tolerated by all patients and had low rates of additional adverse events. There were no EPS reported. Only two patients reported experiencing more sedation [13].

To the best of our knowledge, this is the first trial to compare the effectiveness of quetiapine to the standard medication haloperidol in terms of DRS-R-98 in critically ill patients with hyperactive delirium. This study’s monocentric design might restrict how far the findings can be valid. The sample size calculation was based on the primary outcome only, and this small sample size may make it difficult to detect a mortality difference. No daily assessment for the DRS-R-98 score was planned in our protocol. The study design was liable to selection bias because of the very narrow inclusion and too wide exclusion criteria. There were no specified doses for the study drugs, it was dosing range. Doses were given once daily but at different times of the day. Additionally, most research involving critically ill patients has not been able to identify any direct antipsychotic mortality advantages. All patients in the study were undergoing active treatment, and because the study’s care providers were aware of this, it’s possible that this had an impact on some assessments, such as daily sleeping hours. Although multiple confounding factors may contribute to the duration of ICU stay, further studies should investigate the clinical significance of the decreased ICU stay addressed in this study.

## Conclusions

In the light of these results, quetiapine may be equally as effective as haloperidol in treating the symptoms of hyperactive delirium in critically ill patients, with no mortality benefit.

## Clinical trial registration

ClinicalTrials.gov: NCT05690698.
